# Burden of Mortality from Asbestos-Related Diseases in Italy

**DOI:** 10.3390/ijerph181910012

**Published:** 2021-09-23

**Authors:** Lucia Fazzo, Alessandra Binazzi, Daniela Ferrante, Giada Minelli, Dario Consonni, Lisa Bauleo, Caterina Bruno, Marcella Bugani, Marco De Santis, Ivano Iavarone, Corrado Magnani, Elisa Romeo, Amerigo Zona, Mariano Alessi, Pietro Comba, Alessandro Marinaccio

**Affiliations:** 1 Department of Environment and Health, Istituto Superiore di Sanità, 00100 Roma, Italy; caterina.bruno@iss.it (C.B.); marco.desantis@iss.it (M.D.S.); ivano.iavarone@iss.it (I.I.); amerigo.zona@iss.it (A.Z.); pietro.comba@iss.it (P.C.); 2 Department of Occupational and Environmental Medicine, Epidemiology and Hygiene, Istituto Nazionale per l’Assicurazione Contro gli Infortuni sul Lavoro, 00100 Roma, Italy; a.binazzi@inail.it (A.B.); marcella.bugani@gmail.com (M.B.); a.marinaccio@inail.it (A.M.); 3 Department of Translational Medicine, Università del Piemonte Orientale, 28100 Novara, Italy; daniela.ferrante@uniupo.it (D.F.); corrado.magnani53@gmail.com (C.M.); 4 Statistical Service, Istituto Superiore di Sanità, 00100 Roma, Italy; giada.minelli@iss.it; 5 Epidemiology Unit, Fondazione IRCCS Ca’ Granda Ospedale Maggiore Policlinico, 20100 Milano, Italy; dario.consonni@unimi.it; 6 Department of Epidemiology, Servizio Sanitario Regionale del Lazio, 00100 Roma, Italy; l.bauleo@deplazio.it (L.B.); e.romeo@deplazio.it (E.R.); 7 Department of Prevention, Ministry of Health, 00100 Roma, Italy; m.alessi@sanita.it

**Keywords:** asbestos, asbestos-related diseases, burden of mortality, mesothelioma, lung cancer, asbestosis

## Abstract

Asbestos is one of the major worldwide occupational carcinogens. The global burden of asbestos-related diseases (ARDs) was estimated around 231,000 cases/year. Italy was one of the main European asbestos producers until the 1992 ban. The WHO recommended national programs, including epidemiological surveillance, to eliminate ARDs. The present paper shows the estimate of the burden of mortality from ARDs in Italy, established for the first time. National standardized rates of mortality from mesothelioma and asbestosis and their temporal trends, based on the National Institute of Statistics database, were computed. Deaths from lung cancer attributable to asbestos exposure were estimated using population-based case-control studies. Asbestos-related lung and ovarian cancer deaths attributable to occupational exposure were estimated, considering the Italian occupational cohort studies. In the 2010–2016 period, 4400 deaths/year attributable to asbestos were estimated: 1515 from mesothelioma, 58 from asbestosis, 2830 from lung and 16 from ovarian cancers. The estimates based on occupational cohorts showed that each year 271 deaths from mesothelioma, 302 from lung cancer and 16 from ovarian cancer were attributable to occupational asbestos exposure in industrial sectors with high asbestos levels. The important health impact of asbestos in Italy, 10–25 years after the ban, was highlighted. These results suggest the need for appropriate interventions in terms of prevention, health care and social security at the local level and could contribute to the global estimate of ARDs.

## 1. Introduction

Asbestos is one of the most relevant and widespread occupational carcinogens worldwide. There is much evidence that exposure to asbestos causes malignant mesothelioma and lung, larynx and ovarian cancers [1]. A positive association of asbestos with pharynx, stomach and colorectum cancers has also been recognized, with a limited body of evidence [1]. Moreover, pleural plaques, pleural fibrosis and asbestosis are caused by asbestos exposure [2]. The global burden of asbestos-related occupational diseases has been estimated at around 231,000 cases/year [3]. The major source of asbestos exposure is in work settings, but the health effects of environmental exposure are also recognized, with estimates of the proportion of total malignant mesothelioma cases ranging between 5 and 20% [4,5].

Europe is the current centre of the asbestos-related diseases (ARDs) burden [6], and Italy has been among the main European producers and users of asbestos for a century, until the ban in 1992. In 2007, the International Labor Organization and World Health Organization recommended national programs for the elimination of ARDs, including the National Asbestos Profile (NAP), particularly for the countries that still use asbestos, and to prevent ARDs arising from exposure to the various forms of asbestos still in place and as a result of their use in the past, in asbestos-banned countries [7]. In 2017, the Member States of the WHO—European Region—included the elimination of ARDs among the priority actions to achieve the goals of the 2030 UN Agenda for Sustainable Development and committed to implement ARDs surveillance plans, in order to develop national programs to eliminate ARDs [8].

Arachi and colleagues, in a recent paper on the status of NAPs, categorized Italy among the countries with a *proxy* NAP (Category B) and highlighted the high priority of estimating the disease burden attributable to asbestos in both asbestos-banned and non-banned countries [9].

The aim of the present paper is to show the estimate of the burden of mortality from asbestos-related diseases (ARDs) at the population level in Italy, performed for the first time, in light of the need to respond to three main public health questions.

First, quantifying the burden of ARDs would provide an estimate of the health impact of asbestos exposure in a country that has been among the main European producers and users of this mineral for about one century, until the ban in 1992.

Second, setting up a permanent epidemiological surveillance of ARDs would provide national health authorities with scientific data about temporal and spatial patterns of ARDs distribution, thus supporting them in appropriately allocating resources for diagnosing and treating these diseases. Furthermore, a link between national and regional environmental authorities would contribute to priority resource allocation for cleanup and rehabilitation interventions.

Third, the use of cause-specific mortality data, which are available in many countries, would make this approach relatively replicable, with obvious benefits in the transition to asbestos-free development models. The repeatability requires a thorough study of the health information systems of the candidate countries and the implementation of pilot studies, such as the work of Algranti and colleagues in Latin America countries [10].

In addition, these results could contribute to monitor progress toward the elimination of ARDs at the national level and to estimate the worldwide burden of ARDs [9,11].

Malignant mesothelioma being the most specific outcome for asbestos exposure, many countries have already set up ongoing epidemiological surveillance projects of both mortality and incidence of this disease. National mesothelioma registries are currently operating in Australia, France, South Korea and Italy [12]. Mesothelioma mortality is monitored, among others, in the UK [13], US [14], Spain [15], Greece [16], Brazil [17] and Italy [18]. In some countries (Argentina, Brazil, Colombia and Mexico) without an epidemiological mesothelioma surveillance plan, the burden of asbestos-related cancers due to occupational exposure is estimated using information from different datasets on asbestos-exposed workers and levels of exposure [19].

In this framework, the approach applied in the present study to the epidemiological surveillance of ARDs mortality, partly based on available health information flows and partly based on modelling, might be of interest at a global level, both for countries where the use of asbestos has been banned and countries where it is still legal.

## 2. Materials and Methods

The diseases associated with asbestos exposure with sufficient evidence [1] and with available mortality data—mesothelioma, lung and ovarian cancers, and asbestosis—were included in this study. Larynx cancers and non-malignant pleural diseases were not considered, because of their low fatality rates. The estimates for malignant mesothelioma and asbestosis mortality were based on the cause-specific mortality database managed by the Statistical Service of the National Institute for Health (Istituto Superiore di Sanità: ISS) and provided by the Italian National Institute of Statistics (Istat), updated to 2016 (the last year available at the beginning of the investigation). Asbestos-related lung cancer mortality was estimated applying an ad hoc model, based on the pooled estimates of Italian population-based case-control study findings. Furthermore, the results from a study on the pool of the Italian asbestos occupational cohorts were used for estimating asbestos-related lung and ovarian cancer deaths in some economic sectors with high asbestos exposure levels.

### 2.1. Mortality from Mesothelioma and Asbestosis

We analysed national mortality data from malignant mesothelioma (MM, ICD-10 code: C45) and asbestosis (ICD-10 code: J61) included in the ISS database in the time window 2010–2016. The underlying cause of death reported on the death certificate was considered. Gender-specific standardized mortality rates (using the 2013 European population as reference: https://ec.europa.eu/eurostat/web/products-manuals-and-guidelines/-/KS-RA-13-028, accessed on 9 August 2021) and their 95% Confidence Intervals (95% CIs) were computed at the national level and for the 21 Italian Regions and Autonomous Provinces. Temporal trends of the national rates were also reported.

### 2.2. Mortality from Lung Cancer: Estimated Cases

We applied two methods to estimate the asbestos-related lung cancer (ARLC) burden. The first was based on data from four Italian population-based case-control studies. The second utilized Italian occupational cohorts of workers in industrial sectors with high asbestos exposure levels.

#### 2.2.1. Asbestos-Related Lung Cancer Deaths Estimated on the Basis of Italian Population-Based Case-Control Studies

In order to estimate the number of ARLC deaths in the overall population, the results of the Italian population-based case-control studies included in the SYNERGY Project [20] were considered. Fourteen case-control studies on lung cancer from Europe and Canada, including three from Italy, were pooled to study the joint effects of occupational carcinogens, including asbestos and smoking in relation to lung cancer risk [20]. Within each study centre, cases and controls (population or hospital) were drawn from the same source (study-base). A sensitivity analysis stratifying the analyses by type of controls found that ORs in studies with population-based controls were generally higher and more precise. Among the Italian studies considered to estimate of Attributable Fraction in the present investigation, two studies used population-based controls and contributed 92% of the controls. Individual asbestos exposure was estimated on the basis of a quantitative job-exposure matrix applied to individual job histories, collected by face-to-face interview. Misclassification of exposure could have occurred, but it was most likely nondifferential between cases and controls.

To compute the asbestos Attributable Fraction of lung cancer deaths in Italian studies, we considered the reported values of ORs for ever-exposure to asbestos, adjusted for a number of variables, including: age, cigarette pack-years, time since quitting smoking, and ever-employment in jobs known to entail an increased risk of lung cancer (excluding asbestos exposure). For further details, the reader is referred to the original paper of Olsson and colleagues [20].

In the present analysis, we carried out the following steps.

First, asbestos population attributable fractions of lung cancer deaths (AF_p_) were computed on the basis of the odds ratios (ORs) and on the proportion of the exposed cases (P_ec_) in each study and in the pool using the traditional formula, as follows:

AF_p_ = (OR − 1)/OR = AF_e_ × P_ec_ (1)

Subsequently, to estimate ARLCs, AF_p_ was applied to the annual average number of deaths from lung cancers in the Italian population for the period 2010–2016 (ISS database).

#### 2.2.2. Asbestos-Related Lung Cancer Cases Estimated on the Basis of Italian Occupational Cohort Studies

The findings from the Italian study on occupational cohorts [21] were used to estimate ARLC deaths in the industrial sectors with higher exposure to asbestos (asbestos-cement, rolling stock construction and maintenance, shipyards, dockyards and harbours, glassworks, insulation, asphalt roll production, industrial oven construction and mines). The pool included 43 occupational Italian cohort studies, and 51,801 subjects were considered, after the quality control of data. The uploading of follow-up mortality and ascertainment of employment period using common procedures were performed for each subject. Workers employed in different cohorts were identified, and their work histories were merged in pooled analyses. Standardized Mortality Ratio (SMR) was computed on the basis of sex-age-period-cause specific mortality rates of the region where the cohort was located. Pooled SMRs were adjusted by region, age and period and were presented stratified by sex- and time-related variables [21].

The following procedure was used to estimate ARLC deaths in occupational settings considered in the pool study.

The most recent mortality follow-up (to 2013), available for the single cohorts, was used to obtain cases from the two neoplasms (lung cancer and mesothelioma) and the standardized mortality ratio (SMR) in the aforementioned sectors. The SMR was used to calculate the asbestos attributable fraction among the exposed (AF_e_) using the following formula:

AF_e_ = (SMR − 1)/SMR (2)

The AF_e_ was multiplied for the number of lung cancer deaths in each of the selected occupational cohorts to estimate the number of lung cancer deaths attributable to asbestos exposure (ARLC).

Then, the ratio (R) of ARLC to MM was calculated, assuming the MMs were all caused by asbestos:

R = ARLC/MM(3)

Confidence intervals (CIs) were calculated considering only the numerator as a random variable, using a Bayesian bootstrap method.

The ratios (R) specific to the cohorts were used to estimate the burden of ARLC in the same occupational sectors at the Italian population level, using the archive of the Italian National Mesothelioma Register (ReNaM), a permanent surveillance system of mesothelioma incidence [22]. The cases of mesothelioma registered in the same occupational sectors of the selected cohorts were extracted from the ReNaM database (available period 2010–2015), and the percentage (on the overall ReNaM occupationally exposed cases) was applied to the number of mesothelioma deaths in the Italian population (data from ISS). In this way, the burden of MM in each sector out of those most exposed to asbestos was estimated at population level. The ratios (R) calculated above were then applied to such MM deaths, to estimate the ARLC deaths (ARLC = RxMM).

### 2.3. Mortality from Ovarian Cancer: Estimated Cases

Estimates of asbestos-related ovarian cancer deaths (AROC) were calculated using the same procedure used for ARLC, based on occupational cohort studies in asbestos-cement and glassworks industries, which reported results on ovarian cancers. Occupational cohorts in other industrial sectors and population-based case-control studies on ovarian cancer were not available.

### 2.4. Mortality from Asbestos-Related Diseases (ARDs): Annual Estimated Cases

The estimate of deaths from ARDs was based on the numbers of the observed deaths from malignant mesothelioma and asbestosis, drawn from the ISS database, and the estimated cases of lung and ovarian cancers attributable to asbestos exposure.

## 3. Results

### 3.1. Mortality from Malignant Mesothelioma and Asbestosis

**Figure 1:**
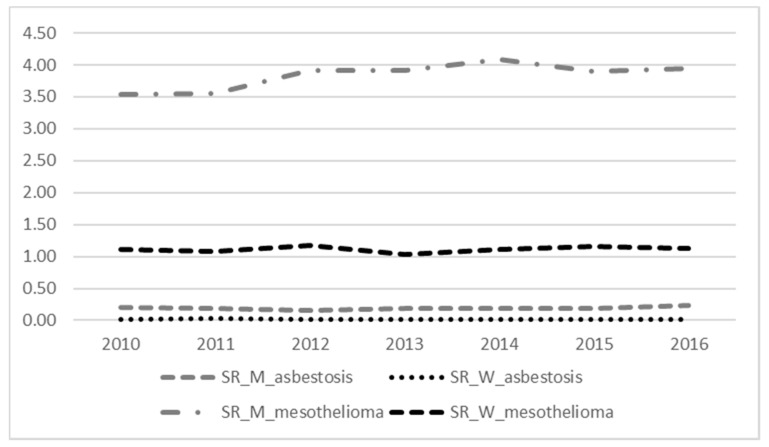
Temporal trend of standardized mortality rates (SR) from malignant mesothelioma and asbestosis (main cause of death) in Italy. Period 2010–2016. (M: men, W: women).

In Italy, in the 2010–2016 period, 10,607 subjects (7660 men, 2947 women) died from MM, corresponding to rates of 3.84 (95%CI: 3.76–3.93) in males and 1.11 (95%CI: 1.07–1.15) in females, per 100,000 person-years. The annual average was 1094 male and 421 female deaths. The annual temporal trend in the considered period was increasing among men and remained stable among women (Figure 1). The distribution of standardized mortality rates from MM by region is shown in Table S1 of the Supplementary Materials. Mortality rates significantly higher than the national average were found in Northern Regions: Piedmont, Lombardy, Friuli Venezia-Giulia and Liguria among men and Piedmont, Lombardy and Liguria among women.

In the same period, 405 subjects (361 men, 44 women) died from asbestosis, corresponding to 0.19 (95%CI: 0.17–0.22) and 0.01 (95%CI: 0.01–0.02) per 100,000 person-years, in the male and female populations, respectively. The temporal trend of rates was stable in both genders (Figure 1). Piedmont, Liguria and Tuscany Regions showed rates of mortality from asbestosis higher than the national average for men; among women, only Piedmont had a rate higher than the national average (Table S2).

### 3.2. Mortality from Lung Cancer: Estimated Cases

Asbestos-related lung cancer cases in the general population, estimated from the results of Italian population-based case-control studies, are reported in Table 1, along with the proportion of the exposed cases, the Odds Ratio (OR) and the attributable fractions (AF_p_) from each study and their pool. The pooled AF_p_ of male lung cancer deaths was 11% (range: 7–12% in the four studies). In women, the mean AF_p_ was 1%, with a low variability among studies.

By applying the pooled AF_p_ to the mean annual deaths from lung cancer in the Italian population between 2010 and 2016 (ISS data), approximately 2718 and 112 ARLC deaths by year were estimated for the male and female populations, respectively. The ranges of deaths estimated from the four studies were 1677–3041 for males and 95–177 for females.

The ratios between ARLC and mesothelioma (R = ARLC/MM) for each working sector of occupational cohorts, along with estimated ARLC and MM deaths in the corresponding ReNaM categories, are reported in Table 2. Overall, 1814 ARLC were estimated in the period 2010–2015. The highest number of males was estimated in the sectors of dockyards and harbours (778), shipyards (467), asbestos-cement industry (229) and glassworks (220), followed by rolling stock construction (79) and ship furniture (9). With regard to females, 16 ARLC deaths were estimated in asbestos-cement industry and in the rolling stock construction sector.

### 3.3. Mortality from Ovarian Cancer: Estimated Cases

Ninety-six ovarian cancer deaths attributable to asbestos exposure, in the period 2010–2015, were estimated in the two considered working sectors (asbestos-cement and glassworks). The ratios between AROC and mesothelioma (R = AROC/MM) with regard to the two working sectors, and the corresponding estimated cases, are reported in Table 3.

### 3.4. Mortality from ARDs in Italy: Annual Estimated Cases in the Period 2010–2016

Overall, around 4400 deaths, approximately 3860 males and 550 females, from asbestos-related diseases by year due to asbestos exposure were estimated in Italy, in the period 2010–2016. These include mean annual observed deaths from mesothelioma (1515) and asbestosis (58) and estimated deaths from lung and ovarian cancers (2830 and 16 cases/year, respectively). While lung cancer deaths could be estimated at population level, ovarian cancer deaths refer to the occupational cohorts of asbestos-cement and glassworks only, for the 2010–2015 period. Annual estimates are not likely affected by the difference of one year in the considered periods.

## 4. Discussion

At present, Italy is one of the countries mostly affected by the worldwide asbestos-related disease epidemic. This condition is a legacy of the massive use of asbestos, mainly from the Second World War to the ban in 1992. During this period, more than 3 million tons of raw asbestos were used in Italy in a wide range of industrial and economic activities [23]. The national consumption of asbestos (production and importation) started decreasing in Italy around ten years after a decrease in the Nordic countries, USA, UK and many other industrialized countries [24].

In addition, the natural presence of asbestos (or asbestos-like) fibres has been repeatedly reported in some areas of the country [25,26].

Clusters of mesothelioma cases among populations residing close to industrial contaminated sites (mostly with asbestos cement plants) were also reported recently [5,27,28,29]. Furthermore, the more recent update of the national surveillance plan based on mortality data, highlighted clusters of deaths from mesothelioma in specific areas of the country and an increasing annual trend in the 2003–2014 period, particularly among the male population [18].

In this context, there is a need to control and contrast the current asbestos-related disease epidemic, as recommended at the international level [7,8]. National epidemiological surveillance of ARDs and the estimate of their burden is a high priority both for asbestos-banned and non-banned countries [8,9]. The present investigation represents an attempt to estimate the magnitude of ARDs in Italy, with the aim to provide evidence of the extent of the public health emergency, to support the effectiveness of the welfare and public insurance system and to facilitate prevention policies and promotion of the awareness of asbestos-exposure health effects.

In Italy, about 4400 deaths per year, approximately 3860 men and 550 women, from asbestos-related diseases were estimated in 2010–2016.

The long period of latency of ARDs, about 40 years for mesothelioma, 10–20 years for asbestosis and around 20 years for lung cancer [20], explains the ongoing ARD epidemic in Italy, 29 years after the asbestos ban. Moreover, the current exposure to asbestos products still in place [30] and/or environmental exposure due to residence near asbestos-like natural fibre sources or contaminated sites could contribute to the current ARD burden. Information on the change in ARDs after the asbestos ban is still limited internationally. In Sweden, a decline in mesothelioma incidence was observed 25–30 years after the prohibition of crocidolite (1975) and all asbestos types (1982) [31]. In the US, the import of asbestos peaked in 1960 followed by a decline; mesothelioma incidence flattened after 1995, with a progressive decline in the observation period until 2005 [32]. Our results are in agreement with the recent prediction of the peak of mortality from malignant pleural mesothelioma in Italy in 2021, suggested by Oddone and colleagues, based on asbestos consumption figures [33]. At the same time, the epidemiological analyses available in the scientific literature, albeit limited, are supportive of the efficiency of the asbestos ban in reducing mesothelioma incidence, as expected.

The present estimates confirm that Italy is still one of the countries most affected by asbestos-related diseases. In the European WHO Region, constituted by 53 countries, 106,180 deaths from ARDs (mesothelioma and asbestosis) in 1994–2010 were estimated [6]. The more recent estimates for Great Britain reported 2369 deaths from mesothelioma in 2019, slightly decreasing with respect to 2012–2018 [13]. In Greece, 53 deaths from mesothelioma per year were observed in the 2001–2003 period [16]. Lopez-Abente and colleagues observed an increase of mortality from mesothelioma in Spain in the 2006–2010 period (1249 deaths) with respect to 1976–1980, and predicted about 1319 deaths for 2016–2020, corresponding to about 264 deaths/year [15]. In the US, a decline has been observed since 1995, with 23,000 cases of mesothelioma (around 639/year) estimated to occur between 2008 and 2024, likely due to asbestos exposure [32].

The past massive use of asbestos and the delay in banning asbestos, as well as the current exposure to residual asbestos sources, could explain the delay in the slope of the ARD burden in Italy. From the end of the Second World War to the asbestos ban in 1992, 3,748,550 tons of raw asbestos were produced, reaching a peak in the period between 1976 and 1980 of about 160,000 tons/year. While in the United States, the United Kingdom and the Nordic countries, asbestos consumption was already decreasing by the early 1960s, in Italy consumption rose in the same period and started to decrease only when it was definitively clear that a ban was necessary. In the 1977–1978 period, asbestos consumption in Italy overtook that of the United Sates, the United Kingdom and the Scandinavian countries, and it remained over 1 kg per capita almost until the ban in 1992, whereas, in other countries, consumption had been below this level for 1–20 years [24]. Even twenty years after the ban, the residual presence of asbestos-containing materials was estimated to be 80 percent of the quantity existing in 1992 [30]; it was calculated that at the beginning of the 2000s, there were still more than 70,000 workers in Italy exposed to asbestos, mainly in construction and in the asbestos-removal sectors [33]. In addition, the natural presence of asbestos or asbestos-like fibres in some areas of the country could represent a source of risk for the general population [25,26].

We considered the mortality for malignant mesothelioma (of all anatomical site), asbestos-related lung and ovarian cancers, tumours associated to asbestos exposure with sufficient evidence [1] and asbestosis. We excluded from the analyses all benign forms, according to the aim of the study, which was focused on mortality. The larynx cancer cases were not computed considering their low fatality levels [34]. The observed deaths from MM and asbestosis were about 1500 and 60 per year, respectively. In addition, 2800 asbestos-related lung and 16 ovarian cancer deaths per year were estimated.

In the analyses by gender, the ratio of male to female ARD cases is around 7:1. The highest M:F ratio was found for ARLC deaths, equal approximately to 24:1. The AFp of deaths from lung cancer estimated among women, on the basis of the pool of Italian case-control studies, is about ten-fold lower than in men (0.01 vs 1.11). The lower levels of asbestos exposure in women at the population level than in men is considered among the possible explanations of the weaker association with lung cancer and asbestos among women and of the difference in the joint effect of asbestos exposure and smoking: multiplicative in men and more than additive in women [20]. The M:F ratio of around 3:1 of mesothelioma deaths, computed on the basis of the Istat database, is in agreement with the ratio by gender of MM incident cases, reported by ReNaM (2.5) [22]. The lower health impact of asbestos, in terms of number of cases, in women with respect to the men, is well documented. The principal source of asbestos exposure occurs in occupational settings, and those with high levels of asbestos exposure are characterized by a lower female workforce. The consistent number of mesothelioma cases in women in Italy is due mainly to the relevant role of non-occupational asbestos exposure and the historical presence of the female workforce in several industrial settings, such as the textile industry (not represented in the present study) [35].

We retrieved all deaths from MM and asbestosis, observed in the national cause-specific mortality database (Istat), taking into account the high etiologic fraction of both diseases due to asbestos (around 80% and 100%, respectively). The etiological fraction attributable to asbestos for lung cancer mortality was estimated from the data of Italian population-based case-control studies and occupational cohort pool. The exact extent of mortality for ovarian cancer due to asbestos exposure is particularly difficult to evaluate, as the level of uncertainty is great. The estimate of AROC in the present investigation was based on the available Italian occupational cohorts, given the lack of Italian population-based case-control studies. The ARLC deaths due to occupational exposure in industrial sectors with high levels of asbestos exposure were estimated at around 302 deaths per year. The use of the data drawn by specific Italian contexts to estimate ARDs is a strength of the present study [36], but the attributable fractions to asbestos of these diseases must be evaluated with caution.

The burden of ARLC is not simply a proportion of MM, and the distribution of exposed workers and people among economic sectors and territories remains a crucial factor to be considered [37]. Furthermore, smoking habits and their synergistic effect with asbestos on the risk of lung cancer is a substantial issue [38,39]. In their pooled analysis of case-control studies, Olsson and colleagues found the joint effect of asbestos exposure and smoking did not deviate from multiplicative among men and more than additive among women [20]. The ARLC presented here were estimated considering the asbestos-attributable proportion of lung cancer deaths based on ORs adjusted for several risk factors, including smoking habits, as detailed in the Methods section.

The ratio of MM deaths to ARLC deaths estimated in this study is about 1:2, higher than the previous estimate for the 1980–2002 period for the Italian context [40]. The estimated ARLC deaths represent 8.4% of all deaths from lung cancer, observed in the same period (2830 ARLC, with respect to 33,524 total lung cancer deaths, by year). This percentage is included in the range estimated in the meta-analysis of European studies [41]. McCormack et al. estimated the ARLC burden using mesothelioma mortality data from asbestos occupational cohorts, calculating the ratios of the absolute number of ARLC to mesothelioma deaths, and the excess lung cancer relative risk to mesothelioma mortality per 1000 non-asbestos-related deaths [42]. For Italy, using these two ratios, they estimated, in percentages, two ranges of ARLC Proportion Attributable Fraction (PAF) in men aged 40–84 years exposed to mixed asbestos fibres: 4.5–5.7 and 7.0–8.7 [42]. Moon et al. estimated PAFs for ARLC by region, using the meta-relative risk of lung cancer from asbestos exposure and asbestos exposure prevalence [36]. PAFs varied in populations of different regions, considering occupational and environmental exposure, with meta-RR respectively equal to 1.88 and 1.06. For Europe, PAFs ranged from approximately 5 to 25%, with an exposure prevalence from 5 to 50% [36]. The results of the present study are in agreement with these estimations.

A key point of our study consists of the methods to identify the ratio between MM and asbestos-related lung cancer. The use of two methods, considering population-based case-control studies and occupational cohorts, is a strength of the present study and allows estimation of ARDs in both the overall population and workers of specific industrial sectors. The use of the findings by occupational pooled cohorts of asbestos-exposed subjects is a strength, considering the large sample size of enrolled subjects and observational period, but this implied a selection of economic sectors [43]. The method based on population case-control studies provides a global estimate in the general population, including all economic sectors, independent from exposure modalities, but the estimate can be affected by the uncertainty deriving from the low accuracy of the exposure definition.

The limitation of the method based on occupational cohorts only is particularly important in the estimation of attributable fraction due to asbestos exposure in ovarian cancer deaths. The exclusion of occupational sectors with relevant asbestos exposure levels where an elevated female workforce is recognized, such as the textile sector [35,44], implies a considerable underestimate of the asbestos-related ovarian cancer deaths obtained in this study.

A further limitation is related to the use of mortality data. The present study is based on death certificates drawn from cause-specific mortality and population databases, provided by the National Institute for Statistics (Istat), available for long-term periods (since 1980) and with a total coverage of the national territory.

The certificates of death from malignant mesothelioma were used as an indicator of the disease burden at the global level [3,45] and in some countries [13,14,15,16,17,18].

The issue of misclassification caused by the use of death certificates in epidemiological surveillance of mesothelioma was raised in the 1970s and 1980s by several authors in different countries [46,47,48,49], including Italy [50,51].

An underestimation of mesothelioma incidence based on death certificates has been reported [52,53,54]. The use of the 10th Revision of ICD Codes, available in Italy since 2003, reduces the possible misclassification by introducing a specific morphological code for mesothelioma [4,53]. In addition, the high mortality rate of the disease mitigates the possible bias, but a remaining effect could not be ruled out, and some prudence in the interpretation of the data is appropriate. A recent study of the causes of death of two mesothelioma case series in the provinces of Trieste and Brescia, including 185 and 90 subjects diagnosed in 1997–2016, respectively, showed a concordance between clinical diagnosis and cause of death of 91% and 92%, respectively. Full concordance was shown in the most recent years of the study (2010–2016), the period used in the present paper [55]. For further discussion of the issue of possible mesothelioma misclassification due to the use of cause of death certification in Italy, the reader may consult the previous papers on surveillance of mesothelioma mortality [56] and comparison with incidence data [21].

Concerning ARLC, the estimate of deaths from lung cancer attributable to asbestos exposure in occupational settings was based on data from the Italian National Registry of Mesothelioma (ReNaM), where the high quality of diagnosis and exposure assessment is well documented. The Italian active mesothelioma surveillance system relies on a long period of activity, near-complete territorial coverage, national standardized procedures for both diagnosis and exposure assessment, data processing and quality control. Sources of incident malignant mesothelioma cases are healthcare institutions that diagnose and treat cases (especially pathology units and lung care and chest surgery wards), and the collected cases are checked with death certificates and data from cancer registries [57]. The complete diagnosis coding system adopted is extensively described elsewhere [58].

The underestimation of the burden of disease in evaluation based on mortality data is particularly important for asbestosis, a low fatality disease.

In Italy, a database of Hospital Discharge (HD) is available, but we did not use it in this analysis to avoid an undercount of the subjects who died with more than one ARD. Considering the database of HD, in Italy 4959 subjects (4603 men, 356 women), about 620 cases/year, were hospitalized from asbestosis (ICD-9 CM code 501) in the 2010–2017 period. In the present study, 58 subjects on average died each year from asbestosis: the high difference of deaths vs hospitalized cases is due to the low fatality of the disease, even if it could represent a co-morbidity with other lethal ARDs. In addition, the difficulty of radiological diagnosis could contribute to an underestimate of the number of subjects affected by asbestosis. A national mortality cohort study of women compensating for asbestosis in Italy showed significant excesses for all causes, all neoplasms, lung, uterine and ovarian cancers, and non-neoplastic respiratory disease. Textile workers, mainly exposed to chrysotile, showed higher SMR for lung cancer and asbestosis. Women employed in the asbestos cement industry, mainly exposed to crocidolite-containing asbestos mixtures, experienced higher mortality for pleural malignancies [59].

The hospitalizations were not computed in the global burden of mortality from ARD estimates, for the above explained reasons, but the burden of asbestosis cases should be considered in the health intervention and surveillance plans.

Incidence data from ReNaM confirmed the global impact of MM in Italy herein estimated by mortality data. Specific surveillance systems of MM incident cases, including anamnestic individual analysis comparable for information completeness, environmental exposure assessment and territorial coverage are scarce and, to the best of our knowledge, currently exist only in Australia, New Zealand, France and South Korea. In the UK, the Netherlands and the Nordic countries, some information is available from other sources [12]. The systematic collection of data about mesothelioma incidence and mortality could significantly improve awareness about asbestos-exposure-related health effects, contributing to the identification of unknown sources of contamination and to the evaluation of the effects of measures for counteracting asbestos exposure. Furthermore, the opportunity of using the early mortality for mesothelioma, as a proxy of a potential environmental exposure to asbestos in childhood, has to be underlined [60].

Performing ARD epidemiological surveillance plans in order to implement actions to eliminate the ARD epidemic is recommended by ILO-WHO [7]. The National Asbestos Profile, including estimates of ARD deaths, is a useful tool to monitor the ongoing actions to address ARDs and to better address future programs, and helps to increase the awareness of asbestos health effects [9]. The need to have a quantitative estimate of the ARD burden in the country to implement appropriate actions of prevention, healthcare and social welfare for asbestos victims, with a homogenous national approach, was pointed out by the Italian Health Minister. The requirement was made by the Interministerial Board on “Asbestos Health”, which includes governmental institution representatives, researchers, traded unions and workers’ associations.

In an international context, the availability of asbestos-related-disease impact estimates at the national level could support the evaluation of the potential path towards an asbestos ban for all countries.

The experience of many Western countries (including Italy) shows the correct approach for addressing asbestos exposure and the related adverse health effects. It includes the production of epidemiological evidence on asbestos-related health effects, the development of awareness in workers, the general population and authorities about the public health risks of asbestos use and the production of evidence on the social, economic and health advantages of asbestos bans.

## 5. Conclusions

Although considering the limitations and the uncertainty of the estimates, our study provides evidence of an important continuing burden of asbestos-related diseases in Italy, despite the asbestos ban in 1992. The results could contribute to the estimate of ARDs at the global level. The strategic importance of epidemiological surveillance of ARDs must be underlined to address the necessary actions towards eliminating ARDs, as recommended by international organizations and governments. The Italian experience could represent a model for countries that are intending to stop the use of asbestos. The effectiveness of prevention policies, social security, welfare and public health system activities, including psychological support, can benefit from the systematic monitoring of the ARD burden.

## Figures and Tables

**Table 1:** Estimated number of asbestos-related lung cancer deaths (ARLC) by year, by case-control studies and their pooled analysis and by gender. Period: 2010–2016.

Gender	Case-Control Study	Proportion of Exposed Cases	OR (95% CI)	AFp	ARLC/Year *
Males	EAGLE (Lombardy)	0.40	1.45 (1.21–1.74)	0.12	3041
	ROMA	0.50	1.16 (0.78–1.72)	0.07	1677
	TORINO-VENETO	0.57	1.21 (1.00–1.48)	0.10	2430
	POOL	0.47	1.31 (1.16–1.49)	0.11	2718
Females	EAGLE (Lombardy)	0.11	1.11 (0.67–1.86)	0.01	95
	ROMA	0.03	Not reported		
	TORINO-VENETO	0.16	1.14 (0.55–2.33)	0.02	177
	POOL	0.12	1.12 (0.74–1.70)	0.01	112

* Rounded to the nearest integer. Proportion of exposed cases: exposed cases/overall cases; OR: Odds Ratio; CI: Confidence Interval; AF_p_: asbestos attributable fraction in population; ARLC/year: asbestos-related lung cancer deaths, by year.

**Table 2:** Asbestos-related lung cancer (ARLC) and malignant mesothelioma (MM) deaths estimated in occupational categories, by gender. Period: 2010–2015.

Occupational Cohorts	Gender	R (95% CI)	MM Cases in the ReNaM Occupational Categories (Corresponding to Those of Italian Cohorts)		MM Deaths	ARLC Deaths (95% CI)
			Incident Cases	% of Exposed *		
Shipyards	Males	0.94 (0.18–1.57)	319	7.30	497	467 (90–780)
	Females	-	6	0.92	24	-
Asbestos-cement	Males	1.13 (1.01–1.24)	130	2.98	203	229 (205–252)
	Females	0.17 (0.07–0.25)	23	3.54	94	16 (6–23)
Crocidolite miners	Males	0.08 (0.00–0.46)	4	0.09	6	0.5 (0.0–2.9)
	Females	-	1	0.15	4	-
Ship furniture	Males	1.21 (0.00–2.44)	5	0.11	8	9 (0–19)
	Females	-	0	0.00	0	-
Dockyards and harbours	Males	2.71 (1.86–3.35)	184	4.21	287	778 (532–961)
	Females	-	1	0.15	4	-
Asphalt rolls production	Males	0.00	0	0.00	0	-
	Females	-	0	0.00	0	-
Rolling stock construction	Males	0.26 (0.00–0.60)	196	4.49	305	79 (0–182)
	Females	1.3 (0–4.21)	3	0.46	12	16 (0–51)
Glassworks	Males	1.91 (0.00–4.11)	74	1.69	115	220 (0–474)
	Females	-	17	2.62	69	-

* Percentage of the overall ReNaM occupationally exposed cases. R: Ratios of ARLC and MM estimated for working sectors of Italian cohorts. ReNaM: National Register of Mesothelioma. CI: Confidence Interval.

**Table 3:** Asbestos-related ovarian cancer (AROC) and malignant mesothelioma (MM) deaths estimated in occupational categories. Period: 2010–2015.

Occupational Cohorts	R (95% CI)	MM Cases in the ReNaM Occupational Categories (Corresponding to Those of Italian Cohorts)		MM Deaths	AROC Deaths (95% CI)
		Incident Cases	% of Exposed		
Asbestos-cement	0.07 (0–0.12)	23	3.54	94	7 (0–12)
Glassworks	1.28 (0–2.18)	17	2.62	69	89 (0–151)

R: Ratios of AROC and MM deaths estimated for working sectors of Italian cohorts; ReNaM: National Register of Mesothelioma; CI: Confidence Interval.

## Supplementary Materials

The following are available online at www.mdpi.com/article/10.3390/ijerph181910012/s1, Table S1: Mortality from malignant mesothelioma (ICD-10 code: C45), by Region and gender. Period 2010–2016. Table S2: Mortality in Italy from asbestosis (ICD-10 code: J61), by Region and gender. Period: 2010–2016.
