# Supplementary material for: Burden of Mortality from Asbestos-Related Diseases in Italy
Source: Int J Environ Res Public Health. 2021 Sep 23;18(19):10012. doi: 10.3390/ijerph181910012 (PMC8508095; doi:10.3390/ijerph181910012)
Supplement: Supplementary file 1 [file ijerph-18-10012-s001.zip › Table S1.pdf]

Table S1. Supplemental material. Mortality from malignant mesothelioma (ICD-10 code: C45), by Region and gender. Period 2010-2016.

REGION	Men		Women	
	**Cases**	**SR (95% CI)**	**Cases**	**SR (95% CI)**
Piedmont	954	5.84 (5.48-6.24)	497	2.33 (2.13-2.56)
Aosta Valley	20	4.47 (2.73-7.65)	10	1.76 (0.83-3.50)
Lombardy	1737	5.48 (5.22-5.75)	927	2.10 (1.96-2.24)
Bolzano	22	1.42 (0.89-2.36)	12	0.62 (0.32-1.11)
Trento	32	1.95 (1.33-2.89)	18	0.79 (0.46-1.30)
Veneto	585	3.67 (3.38-3.99)	219	1.04 (0.90-1.19)
Friuli-Venezia Giulia	329	7.31 (6.54-8.18)	61	0.98 (0.75-1.29)
Liguria	757	11.65 (10.83-12.54)	183	1.90 (1.63-2.23)
Emilia-Romagna	605	3.80 (3.51-4.13)	220	1.05 (0.91-1.21)
Tuscany	457	3.26 (2.96-3.58)	140	0.75 (0.63-0.90)
Umbria	105	3.18 (2.59-3.88)	14	0.29 (0.15-0.54)
Marche	155	2.74 (2.32-3.23)	50	0.70 (0.52-0.94)
Latium	359	1.95 (1.75-2.17)	157	0.64 (0.55-0.76)
Abruzzo	61	1.32 (1.01-1.73)	21	0.36 (0.22-0.57)
Molise	20	1.72 (1.05-2.73)	9	0.67 (0.30-1.39)
Campania	418	2.56 (2.32-2.84)	121	0.58 (0.48-0.69)
Apulia	368	2.86 (2.58-3.18)	111	0.68 (0.56-0.83)
Basilicata	28	1.42 (0.94-2.13)	6	0.29 (0.11-0.68)
Calabria	95	1.50 (1.21-1.85)	31	0.39 (0.26-0.56)
Sicily	421	2.69 (2.44-2.97)	104	0.52 (0.42-0.63)
Sardinia	132	2.38 (1.99-2.85)	36	0.53 (0.37-0.75)
**ITALY**	**7,660**	**3.84 (3.76-3.93)**	**2,947**	**1.11 (1.07-1.15)**

Cases: number of deaths; SR: Standardized Rates per 100,000 (reference: 2013 European population); CI: Confidence Interval.
