# Supplementary material for: Burden of Mortality from Asbestos-Related Diseases in Italy
Source: Int J Environ Res Public Health. 2021 Sep 23;18(19):10012. doi: 10.3390/ijerph181910012 (PMC8508095; doi:10.3390/ijerph181910012)
Supplement: Supplementary file 1 [file ijerph-18-10012-s001.zip › Table S2.pdf]

Table S2. Supplemental material. Mortality in Italy from asbestosis (ICD-10 code: J61), by Region and gender. Period: 2010-2016.

REGION	Men		Women	
	Cases	SR (95% CI)	Cases	SR (95% CI)
Piedmont	56	0.37 (0.28-0.50)	16	0.07 (0.04-0.12)
Aosta Valley	0	0.00 (0.00-0.00)	1	0.13 (0.00-1.28)
Lombardy	33	0.13 (0.08-0.19)	7	0.01 (0.01-0.03)
Bolzano	1	0.07 (0.00-0.73)	0	0.00 (0.00-0.00)
Trento	0	0.00 (0.00-0.00)	2	0.13 (0.08-0.19)
Veneto	4	0.13 (0.08-0.19)	3	0.01 (0.00-0.05)
Friuli-Venezia Giulia	13	0.31 (0.16-0.58)	0	0.00 (0.00-0.00)
Liguria	82	1.31 (1.03-1.65)	2	0.13 (0.08-0.19)
Emilia-Romagna	16	0.11 (0.06-0.19)	3	0.01 (0.00-0.05)
Tuscany	44	0.31 (0.23-0.43)	3	0.01 (0.00-0.06)
Umbria	6	0.18 (0.07-0.45)	0	0.00 (0.00-0.00)
Marche	7	0.14 (0.05-0.31)	0	0.13 (0.08-0.19)
Latium	8	0.04 (0.02-0.10)	0	0.00 (0.00-0.00)
Abruzzo	2	0.04 (0.00-0.20)	1	0.13 (0.08-0.19)
Molise	0	0.00 (0.00-0.00)	0	0.00 (0.00-0.00)
Campania	31	0.13 (0.08-0.19)	1	0.13 (0.08-0.19)
Apulia	15	0.13 (0.07-0.24)	0	0.00 (0.00-0.03)
Basilicata	2	0.10 (0.01-0.48)	1	0.04 (0.00-0.31)
Calabria	1	0.01 (0.00-0.12)	1	0.01 (0.00-0.09)
Sicily	35	0.22 (0.16-0.32)	3	0.01 (0.00-0.04)
Sardinia	5	0.09 (0.03-0.24)	0	0.00 (0.00-0.09)
**ITALY**	**361**	**0.19 (0.17-0.22)**	**44**	**0.01 (0.01-0.02)**

Cases: number of deaths; SR: Standardized Rates per 100,000 (reference: 2013 European population); CI: 95% Confidence Interval.
